# Apathy and Cognitive Test Performance in Patients Undergoing Cardiac Testing

**DOI:** 10.1155/2013/659589

**Published:** 2013-01-21

**Authors:** Lynn Reese Kakos, Michael L. Alosco, Mary Beth Spitznagel, Joel Hughes, Jim Rosneck, John Gunstad

**Affiliations:** ^1^Department of Psychology, Kent State University, Kent, OH 44242, USA; ^2^Department of Psychiatry, Akron City Hospital, Summa Health System, Akron, OH 44304, USA

## Abstract

*Background.* Psychiatric comorbidity is common in patients with cardiovascular disease, with the literature indicating that this population may be at risk for apathy. The current study examined the prevalence of apathy in patients with cardiovascular disease and its relation to aspects of cognitive function. *Methods.* 123 participants from an outpatient cardiology clinic completed a brief neuropsychological battery, a cardiac stress test, and demographic information, medical history, and depression symptomatology self-report measures. Participants also completed the Apathy Evaluation Scale to quantify apathy. *Results.* These subjects reported limited levels of apathy and depression. Increased depressive symptomatology, history of heart attack, and metabolic equivalents were significantly correlated with apathy (*P* < 0.05). Partial correlations adjusting for these factors revealed significant correlations between behavioral apathy and a measure of executive function and the other apathy subscale with a measure of attention. *Conclusion.* Findings revealed that apathy was not prevalent in this sample though associated with medical variables. Apathy was largely unrelated to cognitive function. This pattern may be a result of the mild levels of cardiovascular disease and cognitive dysfunction in the current sample. Future studies in samples with severe cardiovascular disease or neuropsychological impairment may provide insight into these associations.

## 1. Introduction

Cardiovascular disease (CVD) affects nearly 83 million Americans [1] and is the leading cause of disability and mortality [2]. CVD is also associated with heightened risk for neurological disorders (i.e., Alzheimer's disease) and structural brain changes (i.e., white matter hyperintensities [3–5]. Mild deficits in cognitive function are also observed in 15–20% of CVD patients, including frequent impairments in cognitive abilities such as attention, executive functioning, and psychomotor speed [6–9].

Psychiatric comorbidity is also prevalent in persons with CVD with nearly one-fourth of patients with CVD reporting symptoms congruent with a depression diagnosis [10–13]. This pattern is concerning, as CVD patients with depression are at increased risk for mortality [10–12]. Structural brain changes may in part account for depression comorbidity, as many persons with CVD exhibit neuropathology of brain regions commonly involved in depression [4, 14–17]. 

Similar to this high risk of depressive symptoms, recent work also suggests that CVD patients are at risk for apathy [18, 19]. Apathy is distinct from depression [20, 21] and is defined as impaired motivation, a lack of goal-oriented behavior, and diminished cognition [22, 23]. Apathy is associated with poor outcomes, including treatment nonadherence, decreased functional independence, and caregiver distress [24, 25]. It is also likely related to cognitive test performance in patients with CVD. Indeed, apathy is prevalent in many other medical and neurological populations (i.e., Alzheimer's disease, HIV, stroke, and Parkinson's disease) [24, 26–33] and there is growing evidence demonstrating an association between apathy and reduced cognitive function [34–36]. These impairments may be a consequence of neuropathology, as apathy has been linked with frontal lobe damage—a brain region commonly injured in patients with CVD [4, 37–39]. 

The prevalence of apathy in a CVD population has not been empirically examined. Based on the above findings, it is likely that apathy frequently occurs in persons with CVD and is associated with cognitive function [40, 41]. The current study examined the prevalence of apathy among a sample of older adults with CVD undergoing cardiac testing and examined the association between apathy and neuropsychological test performance.

## 2. Materials and Methods

### 2.1. Participants

Participants were recruited from outpatient cardiology clinics. Inclusion criteria required that participants speak English. Exclusion criteria included history of neurological disorder (e.g., dementia, stroke), moderate or severe head injury (defined as greater than 10 minutes loss of consciousness), past or current severe psychiatric illness (e.g., schizophrenia, bipolar disorder), past or current alcohol or drug abuse (as defined by Diagnostic and Statistical Manual of Mental Disorders 4th Edition (DSM-IV) criteria), and a history of learning disorder or developmental disability (as defined by DSM-IV criteria) [42]. Medical history was self-reported and corroborated using medical record review. A total of 123 participants completed the research protocol and these participants averaged 61.42 (SD = 11.55) years of age and were 22.8% female. Demographic and medical characteristics are presented in Table 1.

### 2.2. Measures

#### 2.2.1. Apathy Evaluation Scale

The Apathy Evaluation Scale (AES) was developed as a method of measuring apathy related to brain pathology, in order to discriminate patients with apathy from those without and to distinguish depression from apathy. This measure is comprised of 18 questions on a 4-point Likert scale, thus resulting in scores from 1 to 4 for each item and total scores from 18 to 72, with higher scores representing greater levels of apathy. Internal consistency, test-retest, and interrater reliability indices are satisfactory and range to 0.94 [43]. Further, convergent and discriminant validity scores range to 0.72 [22].

#### 2.2.2. Beck Depression Inventory

Depression was assessed using the Beck Depression Inventory-II (BDI-II). The BDI-II is a check-list of depressive symptoms that demonstrates good psychometric properties in persons with medical conditions (i.e., test-retest reliability of *r* = .93 to *r* = 0.96, and an internal consistency of *r* = 0.54 to *r* = 0.74) [44, 45]. BDI-II scores range from 0 to 63 with higher scores indicative of greater symptomatology.

#### 2.2.3. Neuropsychological Measures

A brief neuropsychological battery was administered to all participants to assess current levels of cognitive function. All measures exhibit strong psychometric properties and they are commonly used in clinical practice and research. The following measures were administered to the participants in the current sample to assess global cognitive function, attention, executive function, memory, and visuospatial abilities:

- Modified Mini-Mental Status Exam (3MS) [46],
- Trail Making Tests A and B (TMT A and B) [47, 48],
- Rey-Osterrieth Complex (Rey-O) Figure Test Copy and Delayed Recall [49],
- Ruff 2 and 7 Test—The Automatic Detection Accuracy (ADA) and Controlled Search Accuracy (CSA) [50].

#### 2.2.4. Cardiovascular Fitness

Metabolic equivalents (METs) is a stress test measure that estimates cardiac workload. METs were obtained from a standardized symptom limited volitional maximal treadmill stress test (GXT). The exercise protocol was comprised of an increase in mill elevation every 60 seconds that estimates a relative increase in workload of 15%, holding speed constant. Testing also utilized an increase in speed every 3 minutes that also reflects a relative increase in workload of 15% from the previous stage. The test was ended at the patient's request, or if the patient showed clinical signs of cardiovascular, neurologic, or musculoskeletal distress. Peak exercise capacity was approximated from the treadmill speed, grade, and duration and was expressed as METs [51].

#### 2.2.5. Demographic and Medical History

Demographic and medical characteristics were ascertained through self-report and corroborated by a medical record review.

### 2.3. Procedure

All study methods were approved by the appropriate IRB approval and all participants provided written informed consent. Participants completed a brief assessment of apathy, depression, and cognitive functioning, including a cognitive battery and self-report measures. Cardiovascular fitness was assessed with an exercise stress test protocol, providing metabolic equivalents (METs) during maximal exercise. Finally, a medical chart review was conducted. 

### 2.4. Statistical Analysis

Pearson correlations were conducted to determine whether demographic and medical variables were correlated with apathy scores and to define possible covariates. Following this analysis, partial correlations controlling for demographic and medical variables that emerged as significant contributors to apathy were performed to examine the correlation between apathy scores and measures of cognitive function. Finally, Chi-square and independent sample *t*-tests were conducted to determine between group differences on the AES scale for demographic, medical, psychological, and neuropsychological variables.

## 3. Results and Discussion

### 3.1. Sample Characteristics

Overall, participants exhibited relatively preserved levels of cardiovascular fitness, as the sample had an average METs value of 9.85 (SD = 3.09); see Table 1. Additionally, when compared to samples of healthy older adults [25], this sample exhibited low levels of apathy with an AES total score of 26.39 (SD = 6.52). BDI-II scores (M = 4.44, SD = 3.75) also fell within the normal range. In terms of cognitive function, the average performance on most tests fell within normal limits, including the 3MS, the Rey-O Copy, TMT-A, and TMT-B. However, participants exhibited mild levels of impairment on Ruff ADA scores (M = 93.27, SD = 6.70), Ruff CSA scores (M = 90.60, SD = 7.10), and Rey-O Delayed Recall scores (M = 13.39, SD = 6.05); see Table 2.

### 3.2. Medical and Demographic Correlates of Apathy

Higher scores on the BDI-II were associated with higher total apathy scores (*r* = 0.49, *P* < 0.01) and AES subscales of behavioral apathy (*r* = 0.45, *P* < 0.01), cognitive apathy (*r* = 0.45, *P* < 0.01), and other apathy (*r* = 0.52, *P* < 0.01). Similarly, a positive history of past heart attack was also associated with higher cognitive apathy subscores (*r* = 0.20, *P* < 0.05). METs were inversely related to cognitive apathy subscores (*r* = −0.20, *P* < 0.05). Other medical and demographic characteristics were unrelated with AES scores (*P* > 0.05). See Table 3.

### 3.3. Cognitive Correlates of Apathy

Partial correlations adjusting for cardiovascular fitness, BDI-II, and past history of heart attack revealed a significant association between the other apathy subscale score with Ruff ADA (*r* = −0.19, *P* < 0.05) as well as TMT B and the behavioral apathy subscale score (*r* = 0.19, *P* < 0.05). No such pattern emerged for any other cognitive variables (*P* > 0.05 for all); see Table 3.

### 3.4. High versus Low Apathy Groups

Given the low prevalence of apathy in the overall sample, exploratory analyses were conducted to determine potential group differences between those expressing apathy 1.5 standard deviations greater than the mean values from a sample of healthy older adults [25]. More specifically, persons scoring 24 and below (*n* = 60) were compared to those scoring 33 and above (*n* = 22); see Table 4. High and low apathy groups did not differ in demographic or medical variables. However, independent sample *t*-tests revealed that those reporting higher apathy levels exhibited higher BDI-II scores (*t*(80) = −6.147, *P* < 0.01). In terms of cognitive test performance, no between group differences emerged, though a trend toward significance was found for TMT A scores (*t*(80) = −1.95, *P* = 0.06); see Table 4.

Although the current study found that apathy was not prevalent in this sample of persons referred for cardiac testing, higher levels of apathy were associated with greater depression symptoms, a history of heart attack, and poorer cardiovascular fitness. In terms of cognitive function, apathy was correlated with poorer performance on tasks of speeded planning and sequencing and cancellation of visual stimuli. Several aspects of these findings warrant further discussion. 

Average apathy scores in the current sample were lower than the proposed cut off score of 37 and above levels used in neurological samples [52]. In fact, the current sample exhibited apathy scores similar to that found in healthy older adults and just 17.9% of the total sample reported apathy significantly above the mean of that previous study [25]. Several characteristics of the current sample may have contributed to this pattern, including age. Study participants averaged 61 years of age and past work has shown that apathy rates increase with age [25]. Similarly, a younger age would generally suggest that participants had CVD for a shorter duration of time compared to an older sample of CVD patients, thus limiting its potential adverse impact on brain structure. However, no association emerged between age and apathy in the current study, suggesting that there are other possible underlying mechanisms.

Indeed, our findings suggest that CVD severity may offer an alternative explanation as poorer fitness and positive history of heart attack were associated with apathy. This pattern indicates that more severe levels of CVD are likely associated with higher levels of apathy. Further, individuals with more severe forms of CVD have shown more pervasive brain changes (i.e., gray matter loss) [53]. Therefore, the generally small effects in this sample likely involve the relatively mild level of CVD in study participants, as the average METs score in this sample was relatively intact [54]. Future studies should examine apathy levels in persons with more severe forms of CVD, such as heart failure (HF). Persons with HF are at high risk for structural brain changes and depressive symptoms [53, 55], increasing the likelihood of apathy symptoms as well. 

Consistent with expectations, depression scores were closely associated with apathy total and subscale scores. Depression and apathy share many features, frequently cooccur, and can result from structural changes in the brain [56–58]. Interestingly, this strong correlation emerged despite very low levels of depression in this study's participants, as the average BDI-II score was low and well below the mild depression cutoff of 10 [59]. In fact, just 8.8% of individuals had a BDI-II score above this cutoff. At study onset, depression was expected to be higher in this group, as previous research indicates up to 23% of individuals with CVD qualify for a depression diagnosis [60]. This pattern may again be a reflection of the relatively mild level of CVD found in the overall sample and findings might differ in other CVD patient groups (e.g., HF). Future studies should explore this possibility.

Apathy was largely unrelated to cognitive test performance in the current study, which is contrary to past work in other medical populations. For example, one study found that apathy was associated with poorer performance on tasks measuring working memory, learning, and cognitive flexibility in HIV samples [61, 62]. Persons with more severe forms of CVD typically exhibit greater structural brain changes and poorer cognitive test performance [63–66]. As is likely the case with the current sample, persons with mild CVD may not exhibit sufficient cerebrovascular damage to the extent of significant cognitive deficits [62]. Consistent with this notion, average test scores for the sample typically fell within normal ranges compared to established normative data. Future studies are strongly encouraged to further examine the relationship between apathy and cognitive function, particularly in light of the documented association between depression and cognitive function in CVD patients [67].

The current study was limited in several ways. The sample consisted of individuals being referred for evaluation of possible CVD and future studies should enroll participants with more severe CVD, particularly those with HF. This approach would likely produce a sample with higher apathy scores, more severe brain changes, and greater impairments on neuropsychological testing. Other related methodological changes would include selectively targeting older patients, those with known cognitive impairment, and/or CVD patients identified as having significant levels of apathy. Each of these approaches would minimize range restriction and promote understanding of apathy in CVD patients. Finally, the study was restricted to an apathy measure based solely on self-report, yet the addition of collateral report of apathy could enhance the current findings. Collateral report might provide additional insight into this population's range of apathy levels. 

## 4. Conclusion

In summary, low levels of apathy were observed in a sample of persons referred for cardiac testing and apathy was largely unrelated to cognitive function. Future research would benefit from utilizing an older population with more severe CVD to clarify the prevalence of and mechanisms for apathy in this population. Further, utilization of modern techniques assessing brain changes in the CVD population, such as functional MRI (fMRI) or diffusion tensor imaging (DTI), might provide additional insight into the proposed relationship between apathy and cognition in CVD patients. A better understanding of this phenomenon could provide helpful information to health care providers, patients, and those regularly caring for individuals with CVD.

## Figures and Tables

**Table 1:** Descriptive statistics for demographic, psychological, and medical information (*n* = 123).

Demographic variables	
Age, mean (SD)	61.42 (11.55)
Male (%)	77.2
Psychological variables	
BDI, mean (SD)	4.44 (3.75)
AES total, mean (SD)	26.39 (6.52)
Medical variables (% yes)	
Coronary Artery Bypass Graft	11.4
Type 2 diabetes	17.1
Heart attack	31.7
High cholesterol	83.7
Heart failure	5.7
High blood pressure	69.1
METs, mean (SD)	9.85 (3.09)

**Table 2:** Descriptive statistics and average *T* based on sample means for neuropsychological tests (*n* = 123).

	Mean	Standard deviation	*T*-score mean
3MS	95.15	4.38	56
TMT A	32.29	9.46	53
TMT B	83.63	38.85	48
Rey-O Copy	27.75	5.25	43
Rey-O Recall	13.39	6.05	49
Ruff ADA	93.27	6.70	41
Ruff CSA	90.60	7.10	43

*Note. *3MS: Mini-Mental State Exam; TMT A: Trail Making Test A; TMT B: Trail Making Test B; Rey-O Copy: Rey Osterrieth Complex Figure Copy; Rey-O Recall: Rey Osterrieth Complex Figure Delayed Recall; Ruff ADA: Ruff 2 and 7 Automatic Detection Accuracy; Ruff CSA: Controlled Search Accuracy.

**Table 3:** Correlation between demographic, psychological, neuropsychological, and medical variables and apathy scores (*n* = 123).

	Total AES	Emotional AES	Behavioral AES	Cognitive AES	Other AES
Age	0.05	0.05	−0.08	0.06	0.10
Gender	−0.12	−0.14	−0.05	−0.11	−0.10
Type 2 diabetes	0.11	0.03	0.07	0.14	0.05
Heart attack	0.17	0.11	0.07	0.20*	0.14
Heart failure	−0.10	−0.02	−0.13	−0.12	−0.01
High cholesterol	0.13	0.05	0.10	0.16	0.06
High blood pressure	0.01	−0.04	−0.02	0.05	−0.06
CABG	0.03	0.09	−0.04	0.04	0.03
METs	−0.16	−0.12	0.00	−0.20*	−0.14
BDI	0.49**	0.11	0.44**	0.44**	0.52**
TMT A	0.05	0.09	0.08	0.01	0.00
TMT B	0.11	0.03	0.19*	0.08	0.06
3MS	0.08	0.11	−0.02	0.16	−0.09
Rey-O Copy	0.05	0.07	0.01	0.06	0.02
Rey-O Delayed	0.08	0.11	0.05	0.06	0.04
Ruff ADA	−0.06	−0.02	−0.07	0.02	−0.19*
Ruff CSA	−0.06	−0.04	−0.06	−0.01	−0.12

*Note. * AES: Apathy Evaluation Scale; CABG: Coronary Artery Bypass Graft; METs: metabolic equivalents; BDI: Beck Depression Inventory; 3MS: Mini Mental State Exam; TMT A: Trail Making Test A; TMT B: Trail Making Test B; Rey-O Copy: Rey Osterrieth Complex Figure Copy; Rey-O Recall: Rey Osterrieth Complex Figure Delayed Recall; Ruff ADA: Ruff 2 and 7 Automatic Detection Accuracy; Ruff CSA: Controlled Search Accuracy.

*P < 0.05. **P < 0.01.

**Table 4:** Comparison of low and high apathy groups on demographic, psychological, neuropsychological, and medical variables.

	Test statistic	*P* value
Chi-squares		
Gender	2.08	0.21
Heart attack	1.54	0.28
Heart failure	1.03	0.50
High cholesterol	1.95	0.32
High blood pressure	0.01	1.00
CABG	0.00	1.00
*t*-tests		
Age	−0.67	0.51
METs	1.02	0.31
TMT A	−1.95	0.06
TMT B	−1.58	0.12
3MS	−0.28	0.78
Rey-O Copy	−0.62	0.54
Rey-O Recall	−0.52	0.68
Ruff ADA	0.77	0.44
Ruff CSA	0.57	0.57
BDI-II	−6.15	0.001

*Note. *AES: Apathy Evaluation Scale; CABG: Coronary Artery Bypass Graft; METs: metabolic equivalents; BDI: Beck Depression Inventory; 3MS: Mini Mental State Exam; TMT A: Trail Making Test A; TMT B: Trail Making Test B; Rey-O Copy: Rey Osterrieth Complex Figure Copy; Rey-O Recall: Rey Osterrieth Complex Figure Delayed Recall; Ruff ADA: Ruff 2 and 7 Automatic Detection Accuracy; Ruff CSA: Controlled Search Accuracy.
